# Exploration of Potential Targets and Mechanisms of Fisetin in the Treatment of Non-Small-Cell Lung Carcinoma via Network Pharmacology and In Vitro Validation

**DOI:** 10.1155/2022/2383527

**Published:** 2022-06-13

**Authors:** Junjun Ling, Yuhong Wang, Lihai Ma, Aoshuang Chang, Lingzhan Meng, Liang Zhang

**Affiliations:** ^1^Department of Oncology, Chongqing Hospital of Traditional Chinese Medicine, Chongqing, China; ^2^Affiliated Hospital of Guizhou Medical University, Guiyang, China

## Abstract

**Purpose:**

The morbidity and fatality rates of non-small-cell lung cancer (NSCLC) were high, although a combination of multiple treatments was used. Fisetin, a small flavonoid compound, had shown anticancer activities. Thus, we aimed at exploring the mechanisms of Fisetin in the treatment of NSCLC.

**Methods:**

TCMSP and Swiss target tools were used to screen the targets of Fisetin, and GeneCards was used to collect the genes related to NSCLC. The genes common to Fisetin and NSCLC were obtained by Venn analysis, whose possible functions were further annotated. A “Compound-Target-Disease” network was then constructed and hub genes were filtered. Also, molecular docking was performed to predict the binding abilities between Fisetin and the hub genes. Then, the effects of Fisetin on the expression of hub genes in lung adenocarcinoma cells were preliminarily evaluated in vitro.

**Results:**

A total of 131 genes common to Fisetin and NSCLC were filtered out, which might be enriched in several biological processes including antioxidation, cell proliferation, and various signaling pathways, such as PI3K-Akt and IL-17 signaling pathways. Among them, PIK3R1, CTNNB1, JUN, EGFR, and APP might be the hub genes. Molecular docking indicated the close bond between Fisetin and them. Experiments implied a possible effect of Fisetin on the expression of hub genes in A549 cells.

**Conclusion:**

The present study found a series of novel targets and pathways for Fisetin treating NSCLC. Multiple angles, targets, and pathways were involved in the biological processes, which need to be verified in further experiments.

## 1. Introduction

Lung cancer is a malignant tumor with the leading incidence and mortality rates in the world. Its morbidity and fatality rate is the first among men and the second among women. Non-small-cell lung cancer (NSCLC) accounts for the vast majority of all lung cancers, about 80–85%. Although it has been explored continuously, the mechanisms of its occurrence and development have not been clearly understood [1]. At present, the treatment for NSCLC includes comprehensive treatment such as radiotherapy, chemotherapy, immunotherapy, and targeted therapy, but many patients still have high recurrence and metastasis rates, which greatly affects the prognosis of the patients [2]. Therefore, finding other prevention and treatment strategies has become an urgent problem to be solved in clinics.

Traditional Chinese medicine has unique advantages in tumor treatment, such as low toxicity, minor side effects, and strong anticancer activity. It can clinically reduce the toxic and side effects of radiotherapy and chemotherapy and improve the immunity, the quality of life, and even the prognosis of patients by “supporting righteousness and removing evil” [3]. Fisetin, an important component of traditional Chinese medicine, is a small flavonoid compound. Moreover, it also exists in a large number of vegetables and fruits, such as apples, glucose, and kiwi fruit. Fisetin has a wide range of antibacterial, antioxidant, and antitumor activities, of which antitumor activity has gradually attracted much attention. For instance, Fisetin may attenuate the proliferation and invasion capabilities, modulate cell cycle progression, inhibit cell growth, and trigger apoptosis in various cancer cell lines, such as HT-29, U266, and PC-3M-luc-6 [4]. A number of signaling pathways, such as PI3K/Akt/mTOR and AMPK [5], PAK4 [6], and MEK/ERK signaling pathways [7], might be involved in the biological processes in Fisetin-treated carcinomas. Our previous work found that Fisetin has a significant inhibitory effect on NSCLC cells in vitro and could reverse the drug resistance [8]. However, the anticancer mechanism of Fisetin is still not fully understood and needs to be further studied.

Traditional studies often focus on a single gene or target, ignoring the complexity and systematicness of biological processes. In recent years, the network pharmacology has developed, which integrates the system biology and pharmacology, integrates the biological network and pharmacology, changes the traditional search for a single target to comprehensive network analysis, emphasizes the interaction mode of multicomponents, multitargets, and multipathways, and is especially suitable for predicting the action target and possible mechanism of natural compounds from traditional Chinese medicines or different plants [9].

Therefore, we intended to analyze the possible anticancer mechanisms of Fisetin on NSCLC from multiple levels and angles through network pharmacology and further explore the pharmacological effect and pharmacodynamic material basis of Fisetin from numerous aspects.

## 2. Materials and Methods

### 2.1. Analysis of Pharmacokinetic Parameters of Fisetin

The traditional Chinese medicine systems pharmacology (TCMSP) database was a public pharmacological analysis platform for studying traditional Chinese medicine, which comprised 499 kinds of Chinese herbs registered in the Chinese pharmacopeia with 29,384 ingredients, 3,311 targets, and 837 associated diseases [10]. Moreover, twelve important ADME-related properties such as human oral bioavailability, half-life, drug-likeness, Caco-2 permeability, and blood-brain barrier were provided. Thus, the pharmacokinetic parameters of Fisetin were obtained through the TCMSP database.

### 2.2. Screening Common Gene Targets Related to Fisetin and NSCLC

#### 2.2.1. Screening of the Target Genes of Fisetin

The target genes of Fisetin were obtained in two ways. First, the target genes/proteins of Fisetin were directly obtained from the TCMSP platform; second, the 2D or 3D structure of Fisetin was downloaded from the PubChem website [11] and was then imported into the Swiss target platform (http://www.swisstargetprediction.ch) for predicting the target genes. The cutoff (probability >0) was set as the screening criteria. After the target genes/proteins were obtained, the protein names were transformed to the corresponding gene names through the UniProt database (http://www.uniprot.org) if necessary. Finally, the target genes obtained in the above two ways were combined to form a gene set.

#### 2.2.2. Screening of NSCLC-Related Genes

In order to obtain NSCLC-related genes, we had a search in the GeneCards tool (http://www.genecards.org) [12]. The keyword “nonsmall cell lung cancer” was entered in the search box to obtain the gene related to this disease.

#### 2.2.3. Screening of the Common Gene Targets

In order to obtain the common target genes of Fisetin and NSCLC, we took the intersection of Fisetin-targeted genes and NSCLC-related genes obtained from the above steps. Through the Venn diagram, the common gene targets are obtained in the intersection, which could be considered as the potential target genes of Fisetin in the treatment of NSCLC.

### 2.3. Construction of Protein-Protein Interaction (PPI) Network of the Common Target Genes/Proteins

A PPI network may help us understand the relationship among the common genes. There may be many interactions between the genes/proteins. The GeneMANIA database (http://www.genemania.org) [13] was used to predict and analyze the protein interactions. The tool is a user-friendly web server, which can be used for constructing a composite gene-gene functional interaction network from a gene list. The potential targets of Fisetin on NSCLC were imported into the tool, and “*Homo sapiens*” was selected to construct a PPI network.

The CytoHubba plug-in of the Cytoscape tool was used to calculate the parameters regarding the gene network. The hub genes were identified and sorted by the betweenness values.

### 2.4. Biological Function Annotation of the Common Targets

In order to understand the biological functions of the common target genes, we conducted Gene Ontology (GO) analysis. At the same time, in order to understand the possible enrichment signaling pathways of these genes, we analyzed the pathway enrichment through the Kyoto Encyclopedia of Genes and Genomes (KEGG) analysis.

During the GO analysis, we first downloaded the c5.go.bp.v7.4.symbols.gmt subset from the Molecular Signatures Database as the background, mapped the genes into the background set, and used the R software package clusterProfiler (version 3.14.3) for enrichment analysis to obtain the gene set enrichment results. The minimum gene set was 5, and the maximum gene set was 5000. The false discovery rate (FDR) < 0.05 was considered to be statistically significant.

During KEGG analysis, we used the KEGG rest API to obtain the latest gene annotation of the KEGG pathway as the background, mapped the genes into the background set, and also used the R software package clusterProfiler (version 3.14.3) for enrichment analysis. Similarly, the minimum gene set was set as 5, and the maximum gene set was 5000. FDR < 0.05 was considered statistically significant.

### 2.5. Establishment of “Compound-Target-Disease” Network

To clearly show the possible relationship among Fisetin, NSCLC, and the gene targets, the “Compound-Target-Disease” network interaction model was constructed by using Cytoscape software, in which “node” represented Fisetin or NSCLC or the targets, and “edge” represented the interaction relationships.

### 2.6. Molecular Docking

Molecular docking between Fisetin and the hub genes/proteins was conducted using the SwissDock tool [14]. The 2D structure of Fisetin was obtained and then converted in Mol2 format through OpenBable software. The crystal structures of the hub proteins were obtained from the RCSB PDB database (http://www.rcsb.org) [15], and their ligand molecules and water molecules were then removed by using PyMol software. After the docking was terminated, the results were imported into USCF Chimera 1.14 software for visualization and analysis.

### 2.7. Validation of Key Targets through In Vitro Assays

The human lung adenocarcinoma cell line, the A549 cell line, was purchased from the American Type Culture Collection and conserved in our laboratory. The use of A549 cell lines was approved by our institutional research ethics committee. The cells were cultured in Dulbecco's modified Eagle's medium (DMEM) containing 5% fetal bovine serum in a humidified atmosphere with 5% CO_2_ at 37°C. The cells were divided into two groups: the control group (DMEM-treated) and the Fisetin-treated group (10 *μ*M Fisetin). After intervention for 24 hours, the supernatant was discarded, and the mRNAs of the top hub genes (PIK3R1, CTNNB1, and JUN) were detected by the RT-PCR assay. The total RNA of cells was extracted and then reversely transcribed to generate cDNA. Real-time PCR was conducted with a PikoReal Real-Time PCR system (Thermo Fisher Scientific, Vantaa, Finland). PCRs were conducted. Each sample was repeated in triplicate. GAPDH was used as the internal reference. The primer sequences were listed as follows:

  PIK3R1 (F: TGACTTGCACTTGGGTGACA, R: TGAAAGCGTCAGCCAAAACG)
  CTNNB1 (F: AAAGCGGCTGTTAGTCACTGG, R: CGAGTCATTGCATACTGTCCAT)
  JUN (F: TCCAAGTGCCGAAAAAGGAAG, R: CGAGTTCTGAGCTTTCAAGGT)
  GAPDH: (F: ATTCCACCCATGGCAAATTC, R: GCATCGCCCCACTTGATT)

A Western blot assay was performed to test the protein expressions. In brief, cells were harvested, washed with ice-cold PBS, and lysed with RIPA buffer supplemented with a protease inhibitor. Proteins were running on a 10% SDS-polyacrylamide gel electrophoresis technique and transferred to polyvinylidene difluoride membranes (Roche). Blots were then incubated in fresh blocking solution with an appropriate dilution of the primary antibody at 4°C for 24 h. The sources of antibodies were as follows: c-Jun (JUN) rabbit polyclonal, Beta-Catenin (CTNNB1) rabbit polyclonal, PI 3 Kinase p85 alpha (PIK3R1) mouse monoclonal, and GAPDH rabbit polyclonal (BIOSS, China).

### 2.8. Statistical Analysis

Differences between the groups were analyzed by using Analysis of Variances (ANOVA), and the Student–Newman–Keuls method was used for comparison between the groups. These analyses were performed by using MedCalc software (15.2.2; Mariakerke, Belgium). *P* < 0.05 was considered statistically significant.

## 3. Results

**Figure 1:**
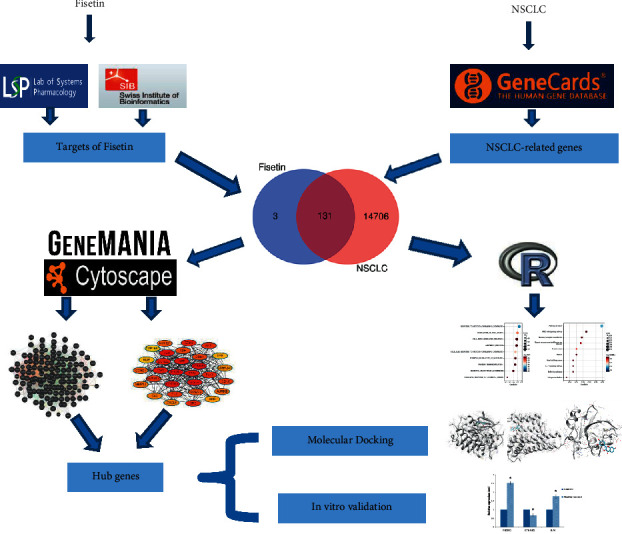
The flowchart of the present study.

Fisetin is a small flavonoid compound that is rich in herbal medicines, vegetables, and fruits. The anticancer activities of Fisetin have attracted much attention. The present study explored the molecular targets and possible mechanisms for Fisetin in the treatment of NSCLC (Figure 1). A total of 131 genes common to Fisetin and NSCLC were screened out, which might be enriched in several biological processes including antioxidation, cell proliferation, and various signaling pathways. PIK3R1, CTNNB1, JUN, EGFR, and APP were identified as the hub genes. Further molecular docking and in vitro assays confirmed the results.

### 3.1. Pharmacokinetic Parameters of Fisetin

**Figure 2:**
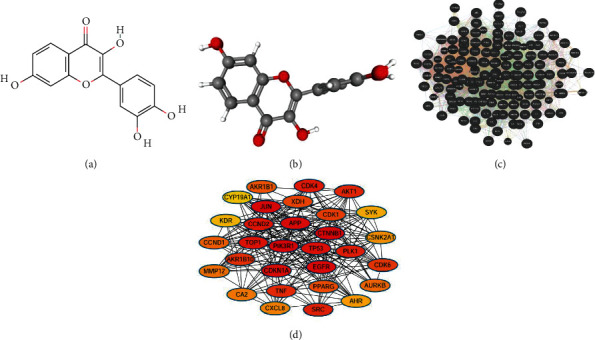
(a) The 2D structure of Fisetin. (b) The 3D structure of Fisetin. (c) The PPI network of the common genes constructed in GeneMANIA. (d) The PPI network of the top 30 genes created by Cytoscape. The nodes represented the genes, and the edges stand for the relationships.

Through the TCMSP database, we obtained the pharmacokinetic parameters of Fisetin. The 2D and 3D structures of Fisetin are shown in Figure 2(a). Its molecular ID is mol013179, molecular formula C_15_H_10_O_6_, relative molecular weight 286.25, the ratio of lipid water partition coefficient (AlogP) 1.77, oral bioavailability (OB) 52.60, blood-brain barrier (BBB) −0.69, and drug-likeness (DL) 0.24.

Among them, OB ≥ 30 and DL ≥ 0.18 were used as the criteria for screening bioactive components in other studies [16, 17]. The pharmacokinetic parameters of Fisetin basically met this standard.

### 3.2. Screening of the Common Targets of Fisetin and NSCLC

First, the targets of Fisetin were screened. Forty-three targets were obtained from the TCMSP database, and 100 targets from the Swiss target database. Then, a total of 134 target genes were obtained after merging these gene sets. Second, a total of 14837 NSCLC-related genes were obtained through the GeneCards tool. Third, 131 genes common to Fisetin and NSCLC were obtained by Venn analysis (Figure 2(b)).

### 3.3. PPI Network of the Common Target Genes of Fisetin and NSCLC

In the process of tumor development, various proteins may interact with each other, thus forming a complex regulatory network and jointly completing various biological processes. The roles of different proteins were varied. Some proteins that have more relationships with other proteins may occupy a greater weight. The genes encoding these proteins are called hub genes.

The network contains 115 nodes, with each node representing a gene/protein and each edge representing the interaction between proteins (Figure 2(c)).

To explore the exact relationships among the genes in the network, Cytoscape software was used to calculate the parameters. The top five genes were listed in the order of betweenness value in Table 1. These genes were at the center of the interaction network and have more interactions with other genes, such as PIK3R1, CTNNB1, JUN, EGFR, and APP (Figure 2(d)).

### 3.4. Function Enrichment Analysis

Go enrichment analysis mainly includes BP (biological process), CC (cellular component), and MF (molecular function). In this study, we selected the most commonly used BP for analysis.

**Figure 3:**
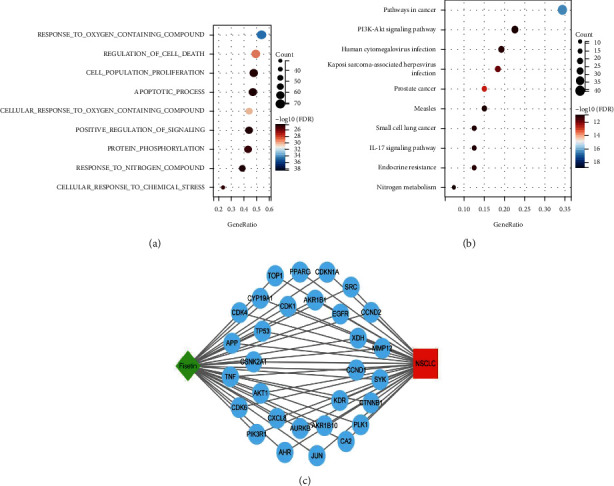
(a) GO analysis of the genes common to Fisetin and NSCLC. (b) KEGG pathway enrichment analysis. (c) The compound-target-disease network. The green diamond represented Fisetin, red square represented NSCLC, and blue circles represented the shared targets.

The GO enrichment results showed that these genes were enriched in 1000 GO terms according to the standard of FDR < 0.05. The top 10 GO terms are listed in Figure 3(a). From these terms, we can know that the genes might be mainly enriched in three major categories of functions: (1) antioxidation, such as response to oxygen containing compounds, cellular response to oxygen containing compounds, and cellular response to chemical stress; (2) cell proliferation and apoptosis, such as cell population proliferation and regulation of cell death; and (3) regulation of signaling pathways, such as positive regulation of signaling and protein phosphorylation.

KEGG pathway enrichment analysis showed that these genes were enriched in 139 pathways (Figure 3(b)). The top 10 signal pathway terms were listed, such as pathways in cancer, PI3K-Akt signaling pathway, IL-17 signaling pathway, small-cell lung cancer, and endocrine resistance.

### 3.5. Establishment of “Compound-Target-Disease” Network Model

A network model was constructed for visualization.  Figure 3(c) shows the network including Fisetin, NSCLC, and the top 30 hub genes.

### 3.6. Molecular Docking

**Figure 4:**
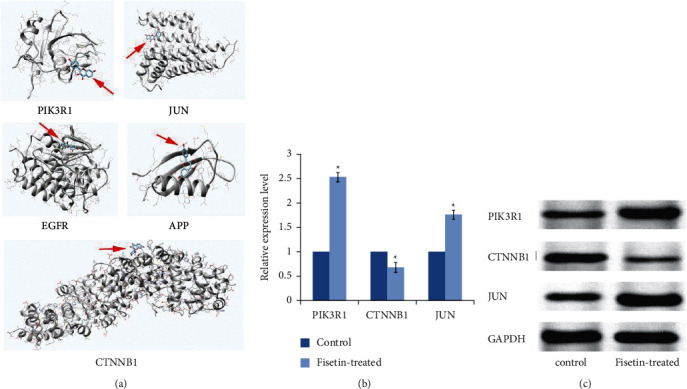
(a) Molecular docking between Fisetin and the top five hub genes (PIK3R1, CTNNB1, JUN, EGFR, and APP). The red arrows pointed to the locations of Fisetin. The ribbons stand for the 3D structures of the proteins. (b) The expression levels of the candidate hub genes (PIK3R1, CTNNB1, and JUN) assessed by qRT-PCR (^*∗*^*P* < 0.05 vs control). (c) The protein expression of PIK3R1, CTNNB1, and JUN detected by western blot.

Docking between the top hub proteins (PIK3R1, CTNNB1, JUN, EGFR, and APP) and Fisetin, respectively, was carried out. If the binding energy was less than zero, it usually indicated that the molecules and ligands could effectively bind with each other. The delta energy for these proteins ranged from −7.61 to −6.31 (Table 1), indicating that Fisetin had a good binding ability with the hub proteins (Figure 4(a)).

### 3.7. Validation of Key Targets

Our previous study has assessed the effects of Fisetin on lung adenocarcinoma cell lines [8]. In the present study, the mRNA expressions of the top three hub genes (PIK3R1, CTNNB1, and JUN) were detected in A549 cells after being treated with or without Fisetin for 24 h. Compared with the control group, respectively, the mRNA expressions of PIK3R1 and JUN in the Fisetin-treated group were significantly increased (*P* < 0.05), while the expression of CTNNB1 was decreased (*P* < 0.05, Figure 4(b)). Additionally, the proteins of the three genes were also detected. The results were in line with the mRNA expressions (Figure 4(c)), indicating that Fisetin administration might have an influence on the expressions of the hub genes.

## 4. Discussion

For discovering drugs, oral bioavailability (OB) is one of the most important pharmacokinetic parameters and an important index to evaluate the effectiveness of drugs entering human circulation. Good OB is the basic premise for compounds to have pharmacological activity. An OB value of greater than 30 is often used as a standard for compound screening. The present study found that the OB value of Fisetin was 52.6, indicating that Fisetin may be easily absorbed into the blood after oral administration, and it thus can conveniently exert biological activity. Drug-likeness (DL) is a physicochemical property and biological characteristic related to good clinical efficacy. The greater the DL value, the safer the compound during absorption and metabolism, suggesting that it is prone to be used as a drug. A DL value of greater than 0.18 is often used as a safety index for drug screening. The DL value of Fisetin was 0.24, indicating that the drug-likeness property of Fisetin was good. Since Fisetin exists in a large number of all kinds of vegetables and fruits in addition to some traditional Chinese medicine, it might be used as a potential dietary agent for disease prevention and treatment.

Fisetin has a variety of biological activities, such as antitumor, anti-inflammatory, and antioxidative stress [18]. For instance, the evidence presented that Fisetin showed the strongest antiaging activity among 10 flavonoids [19]. Fisetin can attenuate lipopolysaccharide-induced acute lung injury by exerting its anti-inflammatory effects [20]. For cancer treatment, Fisetin inhibits the metastatic spread of different cancer cells in tumor-bearing mice by regulating a variety of pathways, such as PI3K/AKT/mTOR, Wnt/*β*-catenin, NF-*κ*B, and TRAIL/TRAIL-R [21]. In addition, Fisetin has the ability to induce autophagic cell death in cancer cells [22]. Thus, the mechanisms of Fisetin in the treatment of cancers were very complex.

Some reports have concerned the effect of Fisetin on lung cancer cells. For example, Fisetin induces apoptosis of the A549 cell line by reducing the expression of c-myc, cyclin-D1, cyclooxygenase-2, Bcl-2, and CXC chemokine receptor 4 [23]. Administration of Fisetin resulted in the decrease of antiapoptotic proteins (such as Bcl-2 and Bcl XL), the increase of proapoptotic proteins (such as Bax and bad), and activation of the caspase-3 signaling pathway in lung cancer cells [24]. However, the published studies in this field were limited, and the molecular mechanisms of Fisetin in the treatment of lung cancer are still poorly understood.

Through the network pharmacology research, we found more gene targets of Fisetin treating NSCLC. The hub genes, including PIK3R1, CTNNB1, JUN, EGFR, and APP, might play an important role in tumor development. For example, PIK3R1 was overexpressed in hepatocellular carcinoma, which promotes cell proliferation and migration capabilities [25]. TP53 is an important tumor suppressor gene, TP53 mutation is a poor prognostic factor for advanced NSCLC, and different mutant exons have different prognostic values. When combined with EGFR mutation, TP53 mutation can more accurately predict the prognosis of patients with advanced NSCLC [26]. CTNNB1 can regulate TP53, thus affecting the progression of lung cancer [27]. As an important part of the JNK signaling pathway, JUN participates in the biological process of different stages of lung cancer [28]. On the basis of the above evidence, we can conclude that the target genes of Fisetin in the treatment of NSCLC may play different roles.

The results of GO analysis suggested that the common target genes were mainly enriched in antioxidation, cell proliferation and apoptosis, and signaling pathway regulation. Pathway enrichment analysis shows that these genes may be involved in various signaling pathways, such as PI3K-Akt signaling pathway, IL-17 signaling pathway, and endocrine resistance. AKT1 is an important signal pathway factor, which participates in all stages of lung cancer occurrence and development [29]; IL-17 can promote angiogenesis through the VEGF pathway, which may indirectly increase the production of NO in the lungs of NSCLC patients and promote the invasion and metastasis of lung cancer [30]. Therefore, these signaling pathways execute different functions in the occurrence and development of lung cancer, while Fisetin may play an intervention role by regulating these signaling pathways.

It is worth noting that some hub genes were downregulated, while others were upregulated after Fisetin-treated lung cancer cells in the process of experimental verification. Because these hub genes have been proved to play a very complex biological role in the process of tumorigenesis and development, hub genes may interact with each other. Also, they may play an antitumor effect by regulating the expressions of other genes. Therefore, the results deserve careful interpretation. For example, the experimental verification part only shows that the administration of Fisetin may change the expression level of these genes in A549 cells. Although molecular docking prediction shows that Fisetin can directly bind to the spatial structure of these hub proteins; in fact, the regulation of Fisetin on them is not necessarily a direct relationship, but it is very likely to be a complex indirect regulation process. Therefore, it is very necessary and important to clarify their relationship in future experimental verification. However, as a prediction tool based on big data, network pharmacology has very important practical significance in guiding the experimental direction, finding the experimental molecular mechanism, and reducing the blindness of exploration.

In the present study, the TCMSP and Swiss target were used to predict the possible targets of Fisetin, while the GeneCards tool was used to obtain the relevant genes of NSCLC. Given that different databases may have different prediction targets due to different algorithms, some target genes may be inevitably missed in the screening process. Thus, the reliability of the obtained genes was inconsistent, which might cause some bias in the results. However, most of the gene targets were obtained due to the use of the mainstream databases for retrieval. It is noted that the low water solubility and rapid metabolism of Fisetin may limit its application in medicine, although it has good oral bioavailability and drug-likeness properties. Thus, novel strategies such as nanocarriers need to be designed and investigated for improving the bioavailability of Fisetin [31].

## 5. Conclusion

The network pharmacology technology used in this study analyzed the potential molecular mechanisms of Fisetin in the treatment of NSCLC from multiple angles. The data suggest that Fisetin may play various roles in treating NSCLC through multiple targets, pathways, and angles. The results also need to be verified in future experiments to further uncover the molecular mechanism of Fisetin in anticancer fields.

## Figures and Tables

**Table 1:** The hub genes from the PPI network and the molecular docking results.

Node_name	Description	Degree	Betweenness	Full Fitness (kcal/mol)	Estimated Δ*G* (kcal/mol)
PIK3R1	Phosphoinositide-3-kinase regulatory subunit 1	152	627.06217	−690.73	−6.31
CTNNB1	Catenin beta 1	143	499.28417	−2550.45	−6.45
JUN	Jun proto-oncogene	127	456.78295	−1712.3	−6.37
EGFR	Epidermal growth factor receptor	145	395.8599	−1791.82	−7.61
APP	Amyloid beta precursor protein	96	376.45816	−334.23	−6.48

## Data Availability

The data used to support this study are available from the corresponding author upon request.

## Abbreviations

NSCLC: Non-small-cell lung cancer
TCMSP: Traditional Chinese Medicine Systems Pharmacology
ADME: Absorption, distribution, metabolism, excretion
PPI: Protein-protein interaction
GO: Gene ontology
KEGG: Kyoto Encyclopedia of Genes and Genomes
FDR: False discovery rate
DMEM: Dulbecco's modified Eagle's medium
ANOVA: Analysis of variances
OB: Oral bioavailability
BBB: Blood-brain barrier
DL: Drug-likeness
BP: Biological process
CC: Cellular component
MF: Molecular function
PIK3R1: Phosphoinositide-3-kinase regulatory subunit 1
CTNNB1: Catenin beta 1
JUN: Jun proto-oncogene
EGFR: Epidermal growth factor receptor
APP: Amyloid beta precursor psrotein.
